# Optimizing cardiac monitoring strategies: patient characteristics associated with different methods of atrial fibrillation detection after stroke

**DOI:** 10.3389/fcvm.2026.1687759

**Published:** 2026-03-26

**Authors:** Kwanju Song, Sujin Koo, Hyun Sun Oh, Jeong Yoon Song, Wookjin Yang, Dong-Wha Kang, Sun U. Kwon, Bum Joon Kim

**Affiliations:** Department of Neurology, Asan Medical Center, University of Ulsan College of Medicine, Seoul, Republic of Korea

**Keywords:** atrial fibrillation, cardiac monitoring, electrocardiography, embolic stroke, embolic stroke of undermined source (ESUS)

## Abstract

**Introduction:**

A variety of cardiac monitoring strategies are currently available, including patch-type monitoring devices and implantable loop recorders (ILR). Identifying patient characteristics associated with atrial fibrillation (AF) detection through different monitoring methods may help inform cardiac monitoring strategies in embolic stroke of undetermined source (ESUS).

**Methods:**

Patients with ESUS who were subsequently diagnosed with AF through continuous stroke-unit electrocardiography monitoring (CEM), Holter monitoring, or ILR were included in the study. Patients were categorized into two groups: short-term monitoring (CEM and Holter monitoring) and long-term monitoring (ILR). Factors associated with the detection of AF through short-term monitoring were investigated.

**Results:**

Among 182 patients with ESUS who were newly diagnosed with AF, AF was detected through CEM (*n* = 92, 50.5%), Holter monitoring (*n* = 67, 36.8%), and ILR (*n* = 23, 12.6%). The prevalence of diabetes was significantly different (40.2% vs. 16.4% vs. 56.5%, *p* < 0.001), while the left atrial diameter (LAD) showed a trend toward significance (41.4 ± 5.9 mm vs. 42.3 ± 6.6 mm vs. 38.4 ± 5.8 mm, *p* = 0.07). Multivariable analysis indicated that diabetes (odds ratio 0.23, 95% confidence interval 0.08–0.63, *p* = 0.004), current smoking (0.29, 0.11–0.79, *p* = 0.015), LAD (1.14, 1.04–1.25, *p* = 0.005), and white blood cell (WBC) count (1.34, 1.07–1.68; *p* = 0.011) were independently associated with the detection of AF through short-term monitoring.

**Discussion:**

Patients with ESUS who have large LAD or elevated WBC counts were more likely to have AF detected during short-term monitoring compared to ILR. Patch-type monitoring devices may be an alternative for these patients prior to the use of ILR.

## Introduction

Cardioembolic stroke, a major subtype of ischemic stroke, necessitates anticoagulation therapy for secondary prevention. Early initiation of this therapy is associated with a reduced risk of stroke recurrence and improved functional outcomes in patients with atrial fibrillation (AF) (1). Given that early AF is correlated with an increased risk of stroke recurrence, timely diagnosis of AF, followed by appropriate anticoagulation, may provide substantial benefits for patients with embolic stroke of undetermined source (ESUS).

Various advanced cardiac monitoring technologies are now available, including telemetry, Holter monitors, and implantable loop recorders (ILR), all of which have proven effective in detecting AF in patients with ESUS. More recently, patch-type cardiac monitoring devices have gained increasing use in clinical practice. While extended monitoring with ILR can capture a greater number of arrhythmic events including subclinical atrial arrhythmias and atrial high-rate episodes whose antithrombotic implications remain uncertain (2), it also entails increased invasiveness and higher costs (3). Selecting the most appropriate monitoring method for individual ESUS patients remains a challenge, particularly as the burden of AF may vary based on the presence of atrial cardiopathy, which is a potential predictor of AF.

In this study, we assessed the characteristics of cardioembolic stroke patients with AF detected through continuous stroke-unit electrocardiography monitoring (CEM), Holter monitoring, and ILR. By investigating the relationship between AF burden and various biological and imaging biomarkers, the study aims to identify patient characteristics associated with AF detection through different cardiac monitoring methods in ESUS.

## Methods

This single-center, retrospective observational study analyzed patients admitted with acute ESUS between January 2017 and December 2023, following the criteria proposed by the Cryptogenic Stroke/ESUS International Working Group (4). In accordance with the center's protocol, all patients with suspected embolic stroke underwent CEM for 2–7 days during hospitalization at the acute stroke unit, followed by Holter monitoring for at least 24 h. ESUS patients diagnosed with AF either during hospitalization or follow-up were included in the analysis. Based on the method of AF detection, patients were classified into three groups: the CEM group, where AF was detected through continuous real-time cardiac monitoring for 2–7 days during hospitalization at acute stroke unit; the Holter group, where AF was detected through Holter monitoring; and the ILR group. Clinical data, including demographic characteristics and stroke risk factors, were obtained from electronic medical records. Stroke severity was assessed at admission using the National Institutes of Health Stroke Scale (NIHSS) score. For patients in whom AF was not detected by CEM and Holter monitoring, ILR was performed at the discretion of the attending clinician. Echocardiography was also conducted on all ESUS patients to measure left atrial diameter (LAD). Laboratory tests included a complete blood count (white blood cell count, hemoglobin, and platelet count), a coagulation profile (prothrombin time and international normalized ratio), as well as measurements of D-dimer and brain natriuretic peptide (BNP) levels. This research was conducted under the approval of the Institutional Review Board (IRB) at our center [approval number: (2024-1074)]. Due to the retrospective nature of the study, the requirement for informed consent was waived by the IRB.

Descriptive statistics were employed to summarize all variables. Continuous variables were reported as means with standard deviations, while categorical variables were presented as counts and percentages. Group comparisons were conducted using the Kruskal–Wallis test for continuous variables and the Chi-square test for categorical variables. Logistic regression analysis was performed by dichotomizing the groups into short-term monitoring (CEM and Holter) and long-term monitoring (ILR). Factors associated with AF detection through short-term monitoring were investigated. Covariates for the multivariable model were selected based on univariable analysis, with variables showing *p* < 0.10 entered into the model. Model calibration and discrimination were assessed using the Hosmer–Lemeshow test and the area under the receiver operating characteristic curve (AUC), respectively. All statistical analyses were carried out using SPSS version 27.0 (IBM Corp., Armonk, NY, USA), with statistical significance defined as a two-sided *p*-value of less than 0.05.

## Results

Over the six-year study period, a total of 5,966 patients were admitted to our center for acute ischemic stroke. Among these, 588 patients (9.9%) were classified as having ESUS. We categorized 182 patients (31.0%) who were diagnosed with AF into three groups: CEM (*n* = 92, 50.5%), Holter monitoring (*n* = 67, 36.8%), and ILR (*n* = 23, 12.6%). Demographic characteristics and stroke risk factors were similar across the groups, with the exception of a lower prevalence of diabetes in patients diagnosed with AF via Holter monitoring. Echocardiographic findings indicated that patients in the CEM (41.4 ± 5.9 mm) and Holter monitoring (42.3 ± 6.6 mm) groups exhibited a trend toward larger LAD compared to the ILR group (38.4 ± 5.8 mm, *p* = 0.07, Table 1). Discharge NIHSS scores were significantly different across three groups [CEM: 4 (interquartile range, IQR: 1–7), Holter: 5 (IQR: 1–8), ILR: 2 (IQR: 2–2), *p* = 0.022).

**Table 1:** Baseline characteristics of the study population.

Variables	CEM (*n* = 92)	Holter (*n* = 67)	ILR (*n* = 23)	*p* value
Age, years	74.7 ± 10.0	74.9 ± 9.8	72.7 ± 9.8	0.77
Male sex	55 (59.8)	39 (58.2)	14 (60.9)	0.98
Hypertension	65 (70.7)	43 (64.2)	7 (30.4)	0.18
Diabetes mellitus	37 (40.2)	11 (16.4)	13 (56.5)	<0.001
Hyperlipidemia	39 (42.4)	30 (44.8)	7 (30.4)	0.50
CAD	19 (20.7)	9 (13.4)	1 (4.3)	0.45
Current smoker	32 (34.8)	21 (31.3)	13 (56.5)	0.09
LAD, mm	41.4 ± 5.9	42.3 ± 6.6	38.4 ± 5.8	0.07
Laboratory tests				
BNP, pg/mL	214.4 ± 303.7	196.0 ± 285.1	139.7 ± 247.3	0.15
D-dimer, mg/L	2.6 ± 5.31	1.4 ± 1.3	0.9 ± 0.8	0.12
CRP, mg/L	0.5 ± 0.9	0.8 ± 1.8	0.5 ± 0.9	0.41
WBC, 10^9^/L	8.1 ± 2.6	8.1 ± 2.7	7.0 ± 1.9	0.20
Initial NIHSS	4 (1–8)	5.5 (2–9.25)	3.5 (1–6.25)	0.42
Discharge NIHSS	4 (1–7)	5 (1–8)	2 (2–2)	0.022
mRS at 3 month	1 (1–3)	1 (0–4)	1 (0–2)	0.32

CAD, coronary artery disease; LAD, left atrial diameter; BNP, brain natriuretic peptide; CRP, C-reactive protein; WBC, white blood cell count; NIHSS, initial National Institutes of Health Stroke Scale; mRS, modified Rankin Scale.

Values are presented as *n* (%), median (interquartile range), or mean ± standard deviation.

Univariable and multivariable logistic regression analyses were performed to identify factors associated with AF detection through short-term monitoring compared to ILR (Table 2). In the univariable analysis, diabetes [odds ratio (OR):0.33, 95% confidence interval (CI): 0.14–0.81, *p* = 0.015], current smoking (OR:0.39, CI: 0.16–0.94, *p* = 0.035), and LAD (OR: 1.10, CI: 1.02–1.19, *p* = 0.015) were significantly associated with short-term monitoring. The multivariable analysis confirmed that diabetes (OR: 0.23, CI: 0.08–0.63, *p* = 0.004), current smoking (OR: 0.29, CI: 0.11–0.79, *p* = 0.015), LAD (OR: 1.14, CI: 1.04–1.25, *p* = 0.005), and elevated white blood cell count (OR: 1.34, CI: 1.07–1.68, *p* = 0.011) were independently associated with AF detection through short-term monitoring methods. The multivariable model demonstrated acceptable calibration (Hosmer–Lemeshow *χ*^2^ = 13.771, *df* = 8, *p* = 0.088) and discrimination (AUC = 0.791, 95% CI: 0.685–0.897). In sensitivity analyses comparing CEM vs. ILR and Holter vs. ILR separately, LAD remained consistently associated with short-term AF detection in both analyses, and WBC count showed a trend toward significance in both comparisons (Supplementary Tables 1 and 2).

**Table 2:** Univariable and multivariable logistic regression analysis of factors associated with atrial fibrillation detection by short-term monitoring strategy.

Variables	Univariable		Multivariable	
	Odds ratio (95% CI)	*p* value	Odds ratio (95% CI)	*p* value
Age	1.02 (0.98–1.07)	0.33		
Male sex	1.08 (0.44–2.63)	0.87		
Diabetes Mellitus	0.33 (0.14–0.81)	0.015	0.23 (0.08–0.63)	0.004
Current smoker	0.39 (0.16–0.94)	0.035	0.29 (0.11–0.79)	0.015
LAD	1.10 (1.02–1.19)	0.015	1.14 (1.04–1.25)	0.005
BNP	1.00 (1.00–1.00)	0.32		
D-dimer	1.53 (0.92–2.54)	0.10		
CRP	1.12 (0.73–1.73)	0.61		
WBC	1.21 (0.99–1.49)	0.07	1.34 (1.07–1.68)	0.011
Initial NIHSS	1.10 (0.94–1.28)	0.23		

LAD, left atrial diameter; BNP, brain natriuretic peptide; CRP, C-reactive protein; WBC, white blood cell count; NIHSS, initial National Institutes of Health Stroke Scale.

## Discussion

In this study, we found that patients with a large LAD were more likely to have AF detected through short-term monitoring methods. It is well established that larger LAD significantly increases the risk of stroke and the likelihood of AF detection (5). Therefore, for ESUS patients with a large LAD, extended cardiac rhythm monitoring using non-invasive methods, such as patch-type cardiac monitoring devices, prior to ILR implantation may warrant consideration in this population. This approach may be particularly beneficial in facilitating early AF detection and the prompt initiation of anticoagulation therapy. An ongoing clinical trial (NCT 05431972) comparing patch-type cardiac monitoring to electrocardiography in ESUS patients with left atrial enlargement may provide further evidence to support this strategy.

The observed relationship between WBC count and the detection of AF during short-term monitoring may be consistent with prior evidence linking systemic inflammation to increased risk of AF (6). Inflammatory processes have been proposed to promote electrical and structural remodeling of the atria, including altered calcium handling and impaired atrial conduction, which may facilitate AF development. However, in the context of acute ischemic stroke, elevated WBC levels may reflect systemic stress responses or acute-phase inflammation, and this possibility warrants cautious interpretation of the observed association.

The inverse associations of diabetes and smoking with short-term AF detection should be interpreted cautiously, as they may reflect differences in AF detection rather than direct causal effects. While diabetes mellitus and smoking are well-established risk factors for stroke, previous studies have shown that they primarily increase the risk of large artery atherosclerosis and small vessel disease (7, 8). It is therefore possible that in these patients, AF developed after the initial stroke caused by other mechanisms, and may have been more paroxysmal or intermittent in nature, making it less likely to be captured during short-term monitoring.

Several limitations of this study should be acknowledged. Its single-center, retrospective design may restrict the generalizability of the findings. Since the study was conducted prior to the widespread use of patch-type cardiac monitoring devices, it is challenging to determine which patients might benefit most from these technologies, and comments regarding their potential role in this population remain exploratory. Additionally, the lower discharge NIHSS scores and lower prevalence of hypertension observed in the ILR group may reflect a selection bias, whereby clinicians were more likely to implant ILR in patients with milder strokes. To evaluate potential selection bias more broadly, we compared baseline characteristics, including diabetes, hypertension, LAD and NIHSS, between ESUS patients who underwent ILR implantation and those who did not. This analysis revealed no significant differences in characteristics between the groups (Supplementary Table 3), suggesting that the decision to proceed with ILR monitoring was not systematically influenced by these clinical parameters. Lastly, patients with AF detected on a 12-lead electrocardiography at the emergency medical center were excluded, as the current study focuses on individuals who may require short- or long-term cardiac monitoring. These patients may not represent the target population.

Despite these limitations, our findings suggest that incorporating cardiac structural parameters, inflammatory markers, and other clinical factors may help optimize both the timing and method of AF detection in ESUS patients. Future research should focus on validating these findings through larger, multicenter studies and investigating whether patients with large LAD or elevated WBC levels might benefit from initial short-term monitoring with patch-type cardiac devices prior to ILR implantation.

## Data Availability

The raw data supporting the conclusions of this article will be made available by the authors, without undue reservation.
