# Supplementary material for: Optimizing cardiac monitoring strategies: patient characteristics associated with different methods of atrial fibrillation detection after stroke
Source: Front Cardiovasc Med. 2026 Mar 26;13:1687759. doi: 10.3389/fcvm.2026.1687759 (PMC13061690; doi:10.3389/fcvm.2026.1687759)
Supplement: Supplementary file 1 [file Table1.docx]

**Supplementary Table 1.** Sensitivity analysis comparing CEM and ILR

|  | **Univariable** |  | **Multivariable** |  |
| --- | --- | --- | --- | --- |
|  | **Odds ratio (95% CI)** | ***p* value** | **Odds ratio (95% CI)** | ***p* value** |
| Age | 1.02 (0.98–1.07) | 0.38 |  |  |
| Male sex | 1.05 (0.41–2.67) | 0.92 |  |  |
| Diabetes Mellitus | 0.52 (0.21–1.03) | 0.16 |  |  |
| Current smoker | 0.41 (0.16–1.04) | 0.06 | 0.33 (0.09–1.24) | 0.101 |
| LAD | 1.09 (1.01–1.18) | 0.033 | 1.15 (1.03–1.30) | 0.018 |
| BNP | 1.00 (1.00–1.00) | 0.30 |  |  |
| D-dimer | 1.47 (0.89–2.42) | 0.13 |  |  |
| CRP | 1.06 (0.61–1.86) | 0.83 |  |  |
| WBC | 1.23 (0.98–1.55) | 0.07 | 1.33 (0.97–1.81) | 0.077 |
| Initial NIHSS | 0.91 (0.88–0.95) | <0.001 | 0.90 (0.86–0.96) | <0.001 |

LAD indicates left atrial diameter; BNP, brain natriuretic peptide; CRP, C-reactive protein; WBC, white blood cell count; NIHSS, initial National Institutes of Health Stroke Scale.

**Supplementary Table 2.** Sensitivity analysis comparing Holter and ILR

|  | **Univariable** |  | **Multivariable** |  |
| --- | --- | --- | --- | --- |
|  | **Odds ratio (95% CI)** | ***p* value** | **Odds ratio (95% CI)** | ***p* value** |
| Age | 1.02 (0.98–1.08) | 0.34 |  |  |
| Male sex | 1.12 (0.42–2.94) | 0.82 |  |  |
| Diabetes Mellitus | 0.15 (0.05–0.43) | <0.001 | 0.25 (0.04–1.49) | 0.13 |
| Current smoker | 0.35 (0.13–0.93) | 0.035 | 0.23 (0.05–1.01) | 0.051 |
| LAD | 1.12 (1.02–1.22) | 0.016 | 1.20 (1.04–1.38) | 0.014 |
| BNP | 1.00 (1.00–1.00) | 0.41 |  |  |
| D-dimer | 1.60 (0.93–2.75) | 0.09 | 2.03 (0.77–5.35) | 0.15 |
| CRP | 1.16 (0.78–1.72) | 0.46 |  |  |
| WBC | 1.20 (0.97–1.49) | 0.09 | 1.34 (0.97–1.85) | 0.072 |
| Initial NIHSS | 0.92 (0.88–0.96) | <0.001 | 0.91 (0.86–0.96) | <0.001 |

LAD indicates left atrial diameter; BNP, brain natriuretic peptide; CRP, C-reactive protein; WBC, white blood cell count; NIHSS, initial National Institutes of Health Stroke Scale.

**Supplementary Table 3.** Baseline characteristics of all ESUS patients according to ILR implantation status

|  | **No-ILR (n=509)** | **ILR (n=79)** | **p value** |
| --- | --- | --- | --- |
| Age | 68 (59-77) | 71.5 (63-78.25) | 0.987 |
| Male sex | 302 (59.3) | 53 (67.1) | 0.362 |
| Hypertension | 305 (59.9) | 49 (62.0) | 0.814 |
| CAD | 92 (18.1) | 17 (21.5) | 0.611 |
| Current smoker | 194 (38.1) | 38 (48.1) | 0.243 |
| Diabetes mellitus | 171 (33.6) | 23 (29.1) | 0.511 |
| LAD | 37 (33-41) | 37 (32.75-40) | 0.641 |
| Initial NIHSS | 2 (1-5) | 2 (1-6) | 0.535 |
| Discharge NIHSS | 2 (0-4) | 2 (0-5) | 0.657 |
| mRS at 3 month | 1 (0-2) | 1 (0-1.5) | 0.172 |

Values are presented as n (%), or median (interquartile range).

CAD indicates coronary artery disease; LAD, left atrial diameter; NIHSS, initial National Institutes of Health Stroke Scale; mRS, modified Rankin Scale.
